# Sunscreen is overwhelmingly promoted on TikTok, but content with misinformation exhibits proportionally high levels of audience interaction

**DOI:** 10.1371/journal.pdig.0001440

**Published:** 2026-06-18

**Authors:** Alessandro Marcon, Marco Zenone, Vincenza Boniface, Cheryl E. Peters, Timothy Caufield

**Affiliations:** 1 Health Law Institute, University of Alberta, Edmonton, Canada; 2 School of Public Policy and Global Affairs, University of British Columbia, Vancouver, Canada; 3 Prevention, Population and Public Health, British Columbia Centre for Disease Control, Vancouver, British Columbia, Canada; 4 Prevention, Screening and Hereditary Cancer Program, BC Cancer, Vancouver, British Columbia, Canada; 5 University of British Columbia, School of Population and Public Health, Vancouver, British Columbia, Canada; The University of Sheffield, UNITED KINGDOM OF GREAT BRITAIN AND NORTHERN IRELAND

## Abstract

This study examined the presence, engagement levels, and characteristics of sunscreen-related misinformation on TikTok. We conducted a content analysis on 971 of the most viewed TikTok videos across the five most popular and relevant sunscreen-related hashtags (#sunscreen, #sunscreenviral, #spf, #sunscreenreview, and #sunprotection). To assess differences in audience engagement (views, likes, shares, and comments) between TikToks that only promoted sunscreen, those that included both promotion and critique, and those that solely critiqued sunscreen, we used Kruskal-Wallis tests and Dunn’s post hoc tests with a Bonferroni adjustment. Most videos promoted sunscreen use (86.8%), typically to prevent skin damage (17.4%), acne (15.3%), aging (11.5%), and cancer (6.1%). Only a small fraction contained critiques (6.0%), with very few asserting sunscreen causes harm (1.5%) or prevents health benefits (1.2%). While differences in view counts were not statistically significant (p = 0.182), TikToks with only critique showed significantly higher engagement in likes (p = 0.0069), shares (p = 0.0028), and comments (p = 0.0012) compared to those with only promotion. Contrary to claims that TikTok is rife with sunscreen misinformation, our findings indicate that such content constitutes a small portion of the most viewed videos. However, these critique focused TikToks, despite being few, consistently generated significantly more audience interaction. This suggests that critical or contrarian content may attract disproportionate attention even when not widely represented. Overall, the dominant narrative on TikTok promotes sunscreen use, and widespread misinformation does not appear prevalent among the platform’s most visible content. Therefore, sunscreen misinformation on TikTok constitutes an area of concern not for the total sum of overarching influence in terms of content production but rather in how strongly some sunscreen misinformation ideas resonated among particular audiences.

## Author summary

This study examined sunscreen-related misinformation on TikTok. We examined 971 of the most viewed TikToks using the five most popular sunscreen-related hashtags (#sunscreen, #sunscreenviral, #spf, #sunscreenreview, and #sunprotection). We found that most videos promoted sunscreen use (86.8%), typically to prevent skin damage (17.4%), acne (15.3%), aging (11.5%), and cancer (6.1%). Only a small fraction contained critiques related to sunscreen (6.0%), with very few claiming sunscreen causes harm (1.5%) or prevents health benefits (1.2%). However, TikToks with only sunscreen-related critique showed significantly higher engagement in likes, shares, and comments compared to those only promoting sunscreen. Contrary to claims that TikTok is rife with sunscreen misinformation, our findings indicate that sunscreen misinformation appears to be in a small proportion of videos. However, these critique focused TikToks, despite being few, consistently generated significantly more audience engagement. This suggests that sunscreen misinformation may attract disproportionate attention. Overall, most TikTok videos promote sunscreen use, and widespread misinformation does not appear prevalent among the platform’s most visible content. Therefore, sunscreen misinformation on TikTok constitutes an area of concern not for the total sum of overarching influence in terms of content production but rather in how strongly some sunscreen misinformation ideas resonated among particular audiences.

## Introduction

Social media use is increasing among all demographics worldwide [1], raising questions of its impact on the public’s understanding and corresponding decision-making related to the health sciences [2,3]. Successfully navigating these online spaces requires high levels of digital health literacy, which relate to one’s ability, capacity, as well as availability of resources to find and evaluate health-related discourse that leads to positive health outcomes [4]. Over the past decade, a growing awareness of online health misinformation correlates with cases of decreased vaccination rates [5], growth of fad diets and unproven biohacking trends [6–8], and a surge in scientifically-unsupported alternative therapies and products [9,10]. Leading academic and public health bodies have identified misinformation as an urgent problem requiring concerted attention [2,11]. It is a problem with heightened relevance in the context of cancer [12–14], which is a leading cause of death worldwide [15]. While cancer causes can be most attributable to family history, genetics, and aging, numerous individual risk-prevention measures can be taken by those fortunate to control lifestyle factors [16]. Individual cancer-risk prevention measures should be informed by accurate science and not influenced by myths, influencer anecdotes, misconceptions, ineffective products, deceptive marketing, or inaccuracies trending on social media or in the public domain [17]. Widespread public prevention measures are also vitally important in the context of public health care systems, where cancer treatments use considerable budgetary funds [18]. It remains essential to counter misinformation so that it does not lead the population towards scientifically unsupported cancer-related activities, products, and therapies or away from accessible evidence-supported prevention measures that can maintain health and save lives.

Skin cancer is primarily caused by exposure to ultraviolet radiation (UV) and consists of melanoma and non melanoma (known as keratinocyte cancer), which is made up of two main subtypes: basil cell carcinoma (BCC), and squamous cell carcinoma (SCC)) [19]. Melanoma is the least common skin cancer type but is the most serious and aggressive, with high potential to spread to other areas of the body, including organs [20]. The risk of melanoma principally increases following multiple high-intensity UV exposures (e.g., sunburns), with the common adage being that five or more sunburns doubles one’s risk [21]. Sunburns during childhood or young adulthood also substantially increases the risk of melanoma as does frequent tanning bed use among younger adults [22]. BCC is the most common form of skin cancer but rarely spreads to other parts of the body. BCC and SCC are both more common but substantially less serious and fatal than melanoma, and typically develop later in life following prolonged but not necessarily high-intensity UV exposure [19]. Advanced SCC, however, can spread to other areas of the body resulting in more serious effects, including death [22]. Like melanoma, intense UV radiation exposures (e.g., sunburns), also can increase the BCC and SCC risk [23]. Broadly, skin cancer risk increases among those with lighter/fairer skin and those with a family history of skin cancer [24]. UV radiation exposure is now a well documented occupational hazard for all professions working outdoors for long periods of time [25].

Skin cancers have high, and growing incidence rates in numerous regions, with “age-standardized incidence rate (ASIR) rising globally (Estimated Annual Percentage Change, EAPC = 1.94%) from 1990 to 2021,” and with notably high ASIR recorded in high-income North America [26]. This is despite the fact that preventative measures are generally affordable and accessible. Avoiding extensive and prolonged UV radiation exposure from the sun or tanning beds can be achieved in a relatively straightforward manner by using protective clothing, applying proper sunscreen/sunblock [27], and avoiding harmful activities such as tanning [28]. In 2024, numerous health bodies observed sunscreen misinformation trending in online public discourse, which included sentiments that sunscreen use was either toxic – and even cancer-causing – or harmfully preventing the benefits of sun exposure (i.e., limiting absorption of Vitamin D). These observed sentiments, in cases expressed by popular celebrities and online influencers, included ideas, for example, that ancestors from generations past spent considerable time in the sun without sunscreen and benefited from such exposure [29]. While not all sunscreen critique is categorically unjustified – in 2021 certain products were recalled for having detectable levels of benzene, an established cancer-causing carcinogen [30,31]– this concern pertained only to a few specific products at a particular time and not broadly to either all non-mineral-based sunscreens or to sunscreen generally as a sun protection tool.

Of critical importance to sunscreen misinformation, there are currently no scientific indications that any ingredient widely used in “chemical” sunscreens (e.g., Oxybenzone) is harmful to one’s health [32,33]. Consumers may prefer a mineral or “natural” sunscreen that blocks UV rays versus a “chemical” sunscreen that absorbs and deactivates rays, but both options are considered safe, and in no circumstance is not using sunscreen a safer option than using a “chemical” product during sun exposure. Rather than an issue of harmful properties, a more pertinent sunscreen issue may be related to SPF accuracy in product labelling as revealed in testing by CHOICE, an Australia consumer advocacy group [34].

Additional concerns have raised about microplastics in sunscreen. Microplastic concerns are not baseless as science continues to assess how microplastic absorption may have negative effects on human health and the environment [e.g., 35]. In the sunscreen context, however, there is no heightened microplastic exposure concern compared to, for example, when humans ingest microplastics via plastic food and drink storage containers [36] or inhale airborne microplastic particles (e.g., synthetic fibres, rubbers, etc. [37]) in indoor and outdoor environments. Public health notices, in response to trending online discourse, observed the possibility of considerable sunscreen related misinformation on TikTok [38–41], one of the world’s most used social media platforms, especially among youth [42]. The observed trending of sunscreen misinformation coincides with a growing body of research showing problematic portrayals of sun protection online, including with intersection of conspiratorial thinking [43–45]. Survey research has similarly found some knowledge accuracy issues related to sun protection and skin cancer [17,46], in particular among younger demographics [47]. Given these concerns, we conducted a systematic assessment of the presence and interaction levels of sunscreen misinformation across the most viewed and engaged with sunscreen content on TikTok. In addition to ascertaining the presence of harmful misinformation, the project mapped out key characteristics in the sunscreen TikTok discourse, including how it was portrayed and engaged with by TikTok audiences.

## Materials and methods

As this research solely analyzed publicly available social media data, ethical review was not required.

### Data collection

On Sept 26^th^, 2024, we conducted an iterative exploratory analysis determining the most used sunscreen related hashtags on TikTok (S1 File). This process involved recording which sunscreen related keywords were used as hashtags on TikToks (e.g., #sunscreen), and in how many posts. We examined associated hashtag lists in the TikTok search bar, and clusters of hashtags used alongside identified relevant hashtags in video captions. This research identified popular and sunscreen related hashtags unique to TikTok, such as “#sunscreenviral” and “#spf.” This same exploratory process was repeated one week later, on October 4^th^, 2024, to determine that no new relevant hashtags could be found, and to assess each hashtag’s percentage change based on total TikTok posts over the one-week time frame. Our analysis found a total of 41 hashtags used in sunscreen-related contexts, 23 of which were frequently used but not sunscreen specific (e.g., #skincareroutine, #skintok, #vitaminD, #dermatology) or not in English (e.g., #protectorsolar). The 18 sunscreen specific hashtags identified (in >800 posts) had been used in more than 3.8 million TikToks. We identified the five most used sunscreen related hashtags of these 18 (#sunscreen, #sunscreenviral, #spf, #sunscreenreview, #sunprotection), which in total had been used in more than 2.9 million TikToks (S2 File). These five sunscreen specific hashtags, accounting for 76% of all posts with sunscreen-related hashtags on our list, were therefore identified as the most popular on TikTok, and which we would use to assemble the TikTok URL data set (S2 File).

We contracted an Apify-hosted data scraper to download a maximum of 1010 of the most viewed publicly accessible TikTok URLs using each of the five hashtags. The goal was to build an initial downloadable data set of 5,000 TikToks, and 1010 was selected as the maximum for each hashtag to provide a small buffer in case of faulty URLS or server time-outs during downloading. Downloaded information included account metadata (e.g., account name, location, etc.) and relevant TikTok data including the total list of hashtags used, upload date, view count, share count, like count, etc. A list of all downloaded variables is available in the supplementary materials (S3 File). To protect the privacy of all TikTok users we followed the Association of Internet Researchers 2019 ethical guidelines on internet research [48], and therefore no account names appear in this manuscript or subsequent research outputs, and no account metadata was used in coding analysis. Given that each hashtag had the potential, and in many cases was observed, to be used in conjunction with the other four hashtags on our list, the total sum of 5,040 downloaded TikToks (8.7 billion views) was compiled into one Google Docs spreadsheet and organized based on view count. With the objective of reaching a finalized data set of approximately 1,000 of the most viewed TikToks, we first removed the 3,040 least viewed TikToks from the list, then removed all additional TikToks from the same user account, save for each unique account’s most viewed TikTok. The objective was to remove the potential for a few individual accounts to skew impressions on the total sum of the data set. Next, we removed all TikToks with no English language text in its caption. The finalized data set consisted of 1,126 TikTok URLs, some of which we assumed would be either non-English or inaccessible for viewing over the coding period, leaving us with an approximate total of 1000 TikToks.

### Data analysis

#### Content analysis.

The primary objective of the analysis was to determine how many TikToks contained critique and how much critique could be categorized as health-related misinformation. For this context, sunscreen misinformation was defined as when TikTok content critiqued sunscreen for either having harmful effects on one’s body or conversely preventing the benefits of sun exposure, which included ideas that sunscreen is unnecessary or ineffective. Additional sunscreen critique (e.g., related to texture or scent) was also captured but not categorized as sunscreen health misinformation. Analysis was not conducted on each individual product profiled to determine whether a product with low SPF was inaccurately promoted for skin protection. Sunscreen misinformation was thus defined from a public health perspective where TikToks, in one form or another, dissuaded its use to the detriment of its valuable health benefits.

To achieve the objective of capturing sunscreen misinformation as well as the overall portrayal of sunscreen across the dataset, we deployed conventional content analysis, which enables the quantification of rich qualitative analysis [49]. Coding frame development followed a multi-stage iterative process, blending a priori understandings of health misinformation and inductive observational analysis that allowed the data under analysis to shape the analytical approach [50]. Working from the defined understanding of sunscreen misinformation for this project, three coders each viewed 100 videos, taking detailed observational notes all video characteristics including narratives, key messages, and recurring themes. These observations resulted in an initial coding frame which was tested on an additional 50 videos by each coder and subsequently modified and finalized based on observations and discussions among coders. An important coding distinction in the finalized coding frame (S4 File) as a result of this process was capturing and distinguishing between broad sunscreen critique (any and all sunscreens as a sun protection tool) versus critique of specific sunscreen brands or products because of their qualities (e.g., a mineral or “natural” sunscreen versus a chemical sunscreen). Socio-demographic coding (gender, ethnicity, status of healthcare professional) was based on visual interpretation, and if possible, other video content (e.g., speech, caption text). It was meant to serve as a broad overview, and not definitive statement, of the TikTok producer demographics in the data set. All data was coded by 2 coders, who highlighted any cases deemed interpretably ambiguous. Following the first round of coding, coders performed an audit of 100 videos on each other’s coding and identified any potential categories of coding variability. All data pertaining to these categories in the entire data set were checked by the original coder, who once again highlighted any cases deemed potentially ambiguous. Finally, each coder performed an additional randomized check of an additional 100 videos, highlighting any perceived coding inconsistencies or cases deemed potentially ambiguous. Coders performed joint coding for all ambiguous cases, reaching coding agreement through consensus.

#### Observational analysis for themes.

Rigorous and systematic thematic analysis [51,52] was not performed in this analysis, however both coders captured topics, themes, and video style trends with note-taking during coding. These observations were shared and discussed following coding completion and are reflected upon in the findings to provide additional context to the descriptive statistics. The themes are thus not detailed at a comprehensive level of saturation to constitute thematic analysis [51–53].

#### Statistical analysis.

We performed a Kruskal-Wallis test followed by a Dunn’s post hoc test with Bonferroni adjustment to determine whether the audience engagement differences observed in mean views, likes, shares, and comments between TikToks with only critique, critique and promotion, and only promotion were statistically significant. We used STATA v.18.0 to perform statistical tests. We did not perform demographic or metadata analysis with regards to TikTok content creators, aside from counting the sum of total posts.

## Results

During coding, 155 of the 1126 TikTok videos (TikToks) were either inaccessible (i.e., non-functioning URLS) or non-English, resulting in a total data set of (N = 971) TikToks, which had amassed over 2.4 billion views, 3.3 million shares, 94.8 million likes, and 728 thousand comments (Table 1). TikToks commonly presented or discussed specific sunscreens and/or sunscreen brands (n = 662, 68.2%), and often promoted specific products (n = 599, 61.7%), in cases by comparing or ranking products (n = 128, 13.2%). Several TikToks included specific purchasing options, such as by offering discount codes in video content or captions (n = 112, 11.5%). While women were present in the majority of TikToks (n = 738, 76.0%), men also had a substantial presence (n = 144, 14.8%). Medical professionals, most noticeably dermatologists, had a presence in approximately 10% of all TikToks, appearing in videos (n = 81, 8.34%) or being referred to by those in videos (n = 15, 1.3%). The TikTok producers were overall diverse, consisting primarily of White (n = 411, 42.3%), Asian (n = 260, 26.8%), and Black (n = 138, 14.2%) peoples (Table 1).

**Table 1:** Content, producer, and engagement characteristics of popular sunscreen related TikToks (N = 971).

Characteristic	n (%)	Characteristic	n(%)
Engagement			
Views	2,470,865,165	Shares	3,353,930
Likes	94,874,802	Comments	728,397
**Broad sunscreen critique**	23 (2.6)	**Sunscreen promotion**	843 (86.8)
Prevents health benefits (incl. unnecessary)	12 (1.2)	General damage (e.g., burning, blistering)	169 (17.4)
Causes health harms	15 (1.5)	Acne/flare-ups related	149 (15.3)
		Aging (incl. wrinkling) related	112 (11.5)
**Specific sunscreen critique**	141(14.5)	Cancer related	59 (6.1)
Health issues (incl. risks)	41 (4.2)		
Incl. chemical vs mineral comparison	45 (4.6)	Promoting solid sunscreen application	260 (26.8)
Health risks based on chemical vs mineral	25 (2.6)	Promoting other sun protection (e.g., clothing)	72 (7.4)
White casting	54 (5.6)		
Greasy/Oily	27 (2.8)	Debunking misinformation	28 (2.9)
Irritating skin	31 (3.2)	Debunk response (incl. vid stitch/text)	13 (1.3)
			
**Total with health issues and harms (in either broad or specific)**	58 (6.0)	**Inclusion of specific product**	662 (68.2)
**Health issues and harms with promotion**	42 (4.3)	**Product promotion**	599 (61.7)
**Health issues and harms without promotion**	16 (1.6)	**Comparing/ranking products**	128 (13.2)
		**Purchase option (e.g., discount code)**	112 (11.5)
**Gender of TikTokers**		**Ethnicity**	
Female	737 (75.9)	White	411(42.3)
Male	144 (14.8)	Asian	260 (26.8)
Non-binary	3 (0.3)	Black	138 (14.2)
Not applicable (no person)	87 (8.9)	Middle Eastern	26 (2.7)
		Other	49 (5.0)
Presence of health professionals		Not applicable (no person)	87 (8.9)
Appearance in TikToks	81(8.3)		
Mentioned in TikToks	15 (1.5)		

### Sunscreen critique

A total of (n = 23, 2.4%) TikToks included broad critique about sunscreen (not product specific) on grounds that it either prevented health benefits (n = 12, 1.2%) or caused health harms (n = 15, 1.5%) (Table 1 and Box 1). A total of (n = 41, 2.6%). TikToks also raised health-risk concerns around specific sunscreen brands or products. Of these 41 videos, 25 attributed health risks to concerns of chemical sunscreens. An additional 20 videos raised issues with chemical vs “natural” sunscreens but did not connect specific health issues to this difference. In total, (n = 58, 6.0%) TikToks included either broad or specific concerns around sunscreen health risks. Many of these videos with critique, however, also included a promotion of sunscreen’s benefits (n = 42, 4.3%). There was a total of (n = 16, 1.6%) TikToks that only detailed health risk concerns around sunscreen without also including sunscreen benefits (Table 1). Regarding non-health risk related critiques, TikToks critiqued specific sunscreens for leaving a white cast (n = 54, 5.6%), having a greasy/oily texture (n = 27, 2.8%), or irritating one’s skin (n = 31, 3.2%) (Table 1).

**Box 1. Examples of sunscreen/sunblock misinformation critiques**

Sunscreen/sunblock preventing benefits (explicit or implied), including sunscreen not necessary

- Preventing tanning/ tanning benefits (creating a beneficial “base”)
- (unprotected) sun exposure is beneficial: sun helps body produce vitamin D, heal itself, produce beneficial neurotransmitters,
- Sunscreen exposure is fine/not dangerous: Thinking of sun damage causes anxiety or reduces enjoyment of being outside: “not necessary to use”, UV exposure damage overrated, constantly pushing for sunscreen (re)application is fear-mongering, sunburns are not dangerous, multiple short exposures (15–30 minutes per time) is fine (“common sense”), sun is not more toxic than sunscreen and harmful food

Sunscreen/sunblock causing harm

- Hormone disrupting potential; “acting as endocrine disruptors”
- Cancer causing; containing “carcinogens”
- Tainting breastmilk
- Containing [harmful] microplastics
- “are toxic”; containing “unsafe” ingredients, e.g., “toxins”
- Damages ecosystems (e.g., coral reefs) and in turn, human health
- Ingestion could cause death

### Sunscreen promotion

The vast majority of TikToks promoted sunscreen use (n = 843, 86.8%), which often contained promotion of specific products (n = 599, 61.7%) (Table 1). A noticeable trend was TikTokers highlighting preferred products and describing their characteristics. TikToks commonly detailed how products felt good on one’s skin, left one’s skin “glowy” or “glowing,” did not interfere with make-up or make-up application (i.e., “pilling”), did not leave an unwanted white cast, or applied easily and absorbed quickly. In promoting sunscreen, TikToks explicitly focused on the health protection and/or benefits sunscreens offer in relation to general damage (e.g., “burning,” “blistering,” “pain,” etc.) (n = 169, 17.4%), acne or skin “flare ups” (n = 149, 15.3%), aging/wrinkling (n = 112, 11.5%), or cancer (n = 59, 6.1%). Thus, while more than 85% of video promoted sunscreen use, cosmetic or application benefits commonly appeared alongside health specific benefits. Observationally, videos did commonly highlight products’ SPF ratings, or UVA and UVB protection capabilities, but notably did so by listing these product attributes along with brand names, brand origins, price and packaging, and descriptions of its feel, smell, or application qualities. While higher SPF was often implicitly or explicitly promoted as necessary, valuable, or even better than lower SPF, explicit critiques of low SPF were not observed nor were critiques of skincare products, for example make-up claiming to offer SPF in non-protective potencies.

Alongside promoting sunscreen generally and specific brands and products, more than a quarter of all videos, stressed the need for sufficient sunscreen application (n = 260, 26.8%), which included advice and tips on applying sufficient quantities (e.g., showing a two-finger application technique), reapplying throughout the day or over specific timelines (e.g., every 2–3 hours), and extending coverage to oft-forgotten places such as hands, lips, ears, neck, etc. An additional 72 TikToks (7.4%) stressed sun protection aside from or in addition to sunscreen, focusing mostly on clothing, such as visors, gloves, sleeves, or masks. Though not numerous, there were a few instances (n = 28, 2.9%) of TikToks countering aspects of perceived sunscreen misinformation (e.g., sunscreen not being important, sunscreen causing harms, tanning not being problematic, etc.). Approximately half of these 28 TikToks (n = 13, 1.3%), contained stitches (copied video segments of others’ TikToks) or other copied information (e.g., text from captions or comments).

### Audience interactions with promotion and health critique TikToks

**Fig 1:**
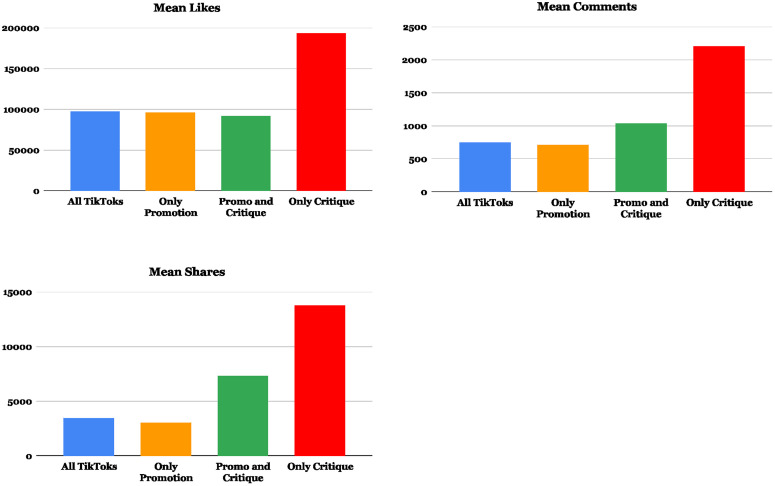
Statistically significant differences in mean likes, shares and comments between all TikToks (n = 971), only Tiktoks with sunscreen promotion (n = 801), Titkoks with sunscreen promotion and critique (n = 42), and Tiktoks with only critique (n = 16).

The four most relevant audience interactions with TikToks are views, likes, shares, and comments. Comparing the mean difference between TikTok with only sunscreen promotion (OP), sunscreen promotion and critique (P&C), and only sunscreen critique (OC) revealed a trend of greater audience engagement in TikToks with critique across the respective mean averages of all audience interactions (Table 2 and Fig 1). The difference in views did not produce a significant Kruskal-Wallis score across the different TikToks (p = 0.182). Following a significant Kruskal-Wallis test for likes, shares, and comments, a Dunn’s post-hoc test with Bonferroni adjustments produced significant differences between OP and OC TikToks for likes (p = 0.0069), shares (p = 0.0028), and comments (p = 0.0012) (Table 2). Thus, TikToks that solely contain sunscreen critique present significant greater audience interaction in terms of likes, shares, and comments in comparison to TikToks that only promote sunscreen. Further, TikToks that include sunscreen promotion and critique also present significantly higher numbers of shares and comments – but not likes – in comparison with TikToks that exclusively promote sunscreen (Table 2 and Fig 1). However, when examining the difference between OC and P&C TikToks for shares and comments, the audience interaction metrics are not significantly different (Table 2). Overall, this suggests that TikToks which include a sunscreen critique, either as the sole focus of the TikTok or in combination with sunscreen promotion, is a driver of increased audience interaction. When critique appears in videos, regardless of promotion, there is an observed and significant boost in audience engagement (Table 2).

**Table 2:** Engagement metrics by observed sunscreen content.

	All TikToks (N = 971)	Only promotion (OP) (n = 801)	% diff*	Promotion and critique (P &C) (n = 42)	% diff*	Only critique (OC) (n = 16)	% diff*	P-value (Kruskal-Wallis test)	Dunn’s post hoc test**
Mean views	2,544,660	2,661,370	4.5	1,826,284	32.9	3,290,496	25.6	0.1817	–
Mean likes	97,708	96,712	1.0	92,447	5.5	193,746	65.6	0.0161	OP-P&C – 1.000 OP-OC – 0.0069 OC-P&C – 0.0122
Mean shares	3,454	3,035	12.9	7,356	72.1	13,794	119.9	<0.000	OP-P&C – 0.0006 OP-OC – 0.0028 OC-P&C – 0.6686
Mean comments	750	716	4.6	1,043	32.7	2,211	98.7	<0.000	OP-P&C – 0.0017 OP-OC – 0.0012 OC-P&C – 0.3906

* difference from mean of all TikToks (N=971).

** with Bonferroni adjustment.

### Additional video elements in sunscreen portrayals

In the overall trend of sunscreen product promotion, TikToks included product evaluation tools and techniques, and educational discourse around sunscreen related terms and definitions. Tools and techniques for evaluating product protection, longevity, and general quality included UV detection sticker dots, UV light testing, and consumer report aps for cellphones. TikToks seldom, if ever, provided critical evaluation of these tools. In educational content, TikToks warned of fake/counterfeit products circulating online, described the differences between UVA and UVB rays, “sunscreen” and “sunblock”, and “chemical” versus “natural,” “mineral,” or “physical” products, with chemical products described as containing ingredients such as oxybenzone, avobenzone, or octinoxate, and natural products described as containing ingredients such as zinc oxide and titanium dioxide. TikTokers often expressed a preference for either chemical or natural products, and in cases, described how chemical sunscreens were harmful for coral reefs. Across all videos, there was a trend of promoting Korean skin care products and brands (see hashtag analysis). Some videos stressed a preference for Korean, or other international brands on grounds that American regulatory bodies have not been as rigorous in updating UV filter standards over the past three decades.

### Hashtag analysis

**Fig 2:**
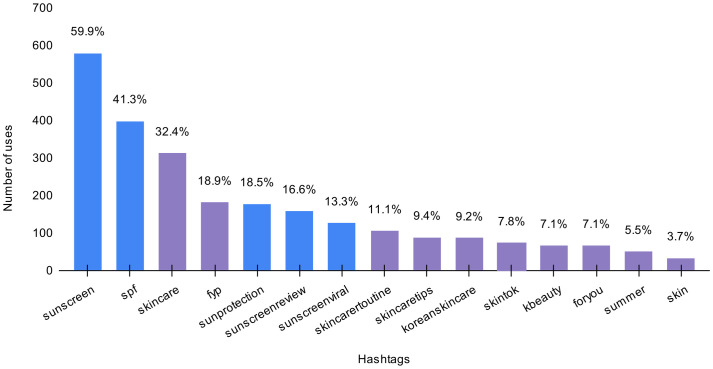
Most used hashtags in Tiktoks (N = 971) with blue bars representing hashtags selected for data collection.

A total of 8,104 hashtags were used in the 971 TikToks, with an average of 8.3 hashtags per TikTok. Of the 8,104 hashtags used, 3,106 were unique. The five hashtags chosen for TikTok downloads were among the most used, with the remaining popular hashtags focused on skincare (e.g., #skincare, #skincareroutine) and general popularity (e.g., #fyp) (Fig 2). This demonstrates how sunscreen content was highly intertwined with general skin and skincare content on TikTok.

## Discussion

This project, contrary to the findings from other research [32], provides strong evidence that popular sunscreen-related TikTok content is not “rife” with sunscreen misinformation that portrays sunscreen as either harmful to one’s health or preventative of sun exposure benefits. Of the 971 most popular and engaged with sunscreen related TikToks, we found only 58 (6.0%) critiquing sunscreen on these grounds. Further, of these 58 videos with critique, the majority (72%) also included sunscreen promotion, leaving only 16 TikToks (1.6%) which critiqued sunscreen without any promotion, thus categorically dissuading its use. This project therefore corroborates findings from earlier media research on sunscreen in the popular press and on X (formerly Twitter), which similarly found a very low, but not completely absent, presence of misinformation [54,55].

However, while sunscreen misinformation constituted only a small proportion of popular sunscreen TikTok content, this small number of TikToks generated substantial and significant levels of audience engagement. Indeed, the 16 (1.6%) TikToks presenting a categorical critique of sunscreen had a significantly higher mean number of shares and likes compared with promotional videos (Table 2). TikToks with critique also generated a considerably greater mean number of comments (Table 2). Analyzing comments on the TikToks was beyond the scope of this project but it would be highly valuable for future research to assess the extent to which TikTok commentary supported or critiqued misleading and inaccurate sunscreen ideas. It may also be valuable to assess whether other TikTok characteristics such as TikTok length could be an influential factor on audience engagement.

Sunscreen misinformation on TikTok therefore constitutes an area of concern not for the total sum of overarching influence in terms of content production but rather in how strongly some sunscreen misinformation ideas resonated among particular audiences. This demonstration of sunscreen misinformation on social media aligns with a growing understanding of the misinformation problem as one that can contain small but highly influential numbers of content producers, whose shock-provoking and “sticky” content can generate considerably and disproportionately greater attention or interaction among some viewers [56,57]. Indeed, misleading and contrarian ideas, for example, that sunscreen is either useless or harmful, incorporates novelty, shock value, and conspiratorial components that trigger emotions and leads to increased viral potential, especially among groups or online communities with a propensity to be intrigued by this type of content [58]. Repeated exposures to this sort of misinformation not only enhances the potential for online viral spreads but increases perceptions of accuracy among all audiences [59]. Thus, counter to critiques of the misinformation problem severity [60], this study adds to the evidence that misinformation does not have to be “rife” to be of potential concern [61]. It is therefore a sound response from public health agencies to immediately and swiftly sound the alarm of an observed misinformation trend rather than idly permit the problematic discourse to foster. At the same time, it remains imperative for public health agencies, wherever possible to increase surveillance capacity to not only observe emerging trends in problematic information but to accurately discern the extent of the burgeoning issues.

Further adding to the potential concern of sun protection content on TikTok is one, evidence of misinformation related to skin cancer, where pro-tanning messages circulate [62], but also, as evident in this research, that sunscreen promotion on the platform is both highly commercialized and highly focused on aesthetics rather than explicit health benefits. Indeed, 62% of all TikToks promoted specific products, 15% promoted sunscreen for acne-related benefits, and 12% promoted sunscreen for reducing the effects of aging. Only 6% of the TikToks explicitly mentioned the benefits of reducing cancer risks. It is worth questioning whether such a prominent focus on skin aesthetics might exacerbate the gender divide around promoting sunscreen’s health benefits [63].

It is not conclusive from this study whether the health benefits of sunscreen are implicitly communicated and collectively understood by TikTokers. For public health, on one hand, the almost unanimous promotion of sunscreen should be celebrated, especially seeing as the promotion was carried out by a diverse body of TikTokers including a not-negligible presence of male content creators (14.8%). On the other hand, this portrayal represents a somewhat missed opportunity to communicate the important science of sunscreen’s vital health benefits. It is a growing challenge for online science communication and initiatives to bolster digital health literacy to compete with content that can easily leverage the advertising power of industry brands and products [64]. It is a challenge requiring ongoing and creative approaches to online discourse that can reach and influence audiences with important health messages and information evaluation strategies needed for positive health outcomes.

## Supporting information

S1 File Iterative exploratory analysis determining popular sunscreen hashtags on TikTok. (DOCX)

S2 File Relevant data for identifying and selecting 5 most used sunscreen related hashtags for data collection. (DOCX)

S3 File Downloaded variables. (DOCX)

S4 File Coding frame. (DOCX)
