# Supplementary material for: Sunscreen is overwhelmingly promoted on TikTok, but content with misinformation exhibits proportionally high levels of audience interaction
Source: PLOS Digit Health. 2026 Jun 18;5(6):e0001440. doi: 10.1371/journal.pdig.0001440 (PMC13278394; doi:10.1371/journal.pdig.0001440)
Supplement: S1 File — (DOCX) [file pdig.0001440.s001.docx]

**S1: Initial exploratory surveillance of “sunscreen” on TikTok**

Initial searches with “sunscreen” on TikTok from September 2024 using a cell phone as well as a laptop presented a high proportion of videos critiquing sunscreen, raising concerns of health harms related to chemical sunscreens as well as general sunscreen use.

Videos included the following ideas:

- Some sunscreens contain harmful chemicals and should not be used by the public
- Harmful sunscreens have pulled from commercial markets by regulatory authorities
- Health influencers talking about never needed to use sunscreen (preventing health benefits)
- Sunscreen should be used with caution / sunscreen is dangerous

Videos included first person (POV) videos but also discussions between various individuals in podcast format.

A quick scan of other noncritical videos:

- People rating sunscreen
- People applying sunscreen
- People ranking sunscreen brands
- People showing skincare routines

Initial observed hashtags:

#sunscreen

#spf

#skincareroutine

#sunscreenviral
