# Supplementary material for: Sunscreen is overwhelmingly promoted on TikTok, but content with misinformation exhibits proportionally high levels of audience interaction
Source: PLOS Digit Health. 2026 Jun 18;5(6):e0001440. doi: 10.1371/journal.pdig.0001440 (PMC13278394; doi:10.1371/journal.pdig.0001440)
Supplement: S2 File — (DOCX) [file pdig.0001440.s002.docx]

**S2: Analysis determining the most used sunscreen related hashtags on TikTok (September-October 2024)**

| Hashtag* | # posts (date 1 – Sept 26) | # of posts (Date 2 – Oct 4) | Growth (% difference) | Inclusion?** | % of total | % of sunscreen | % of 5 selected | Sum |
| --- | --- | --- | --- | --- | --- | --- | --- | --- |
| #skincareroutine | 7,300,000 | 7,400,000 | 1.36 | M | 47.74% |  |  |  |
| #skincaretips | n/a | 2,700,000 | n/a |  | 17.42% |  |  |  |
| #sunscreen | 1,800,000 | 1,800,000 | 0.00 | Y | 11.61% | 47.03% | 61.92% | 2,906,800 |
| #skintok | 751,700 | 777,800 | 3.41 |  | 5.02% |  |  | 75.94% |
| #sunscreenviral | 495,000 | 517,300 | 4.41 | Y | 3.34% | 13.51% | 17.80% |  |
| #tanning | 428,300 | 430,600 | 0.54 | M | 2.78% | 11.25% |  |  |
| #spf | 327,400 | 330,600 | 0.97 | Y | 2.13% | 8.64% | 11.26% |  |
| #sunburn | 193,200 | 193,800 | 0.31 | M | 1.25% | 5.06% |  |  |
| #vitaminD | 175,400 | 179,200 | 2.14 |  | 1.16% |  |  |  |
| #dermatology | 165,400 | 167,300 | 1.14 |  | 1.08% |  |  |  |
| #sunscreenreview | 130,600 | 135,900 | 3.98 | Y | 0.88% | 3.55% | 4.68% |  |
| #sunprotection | n/a | 123,000 | n/a | Y | 0.79% | 3.21% | 4.23% |  |
| #skintips | 104,500 | 105,600 | 1.05 |  | 0.68% |  |  |  |
| #protectorsolar | 102,000 | 103,900 | 1.85 | M | 0.67% |  |  |  |
| #suncream | 89,700 | 90,600 | 1.00 | M | 0.58% | 2.37% |  |  |
| #skincareroutinetips | 71,900 | 72,600 | 0.97 |  | 0.47% |  |  |  |
| #skincarerountines | 56,100 | 58,300 | 3.85 |  | 0.38% |  |  |  |
| #sunscreenisimportant | n/a | 41,600 | n/a |  | 0.27% | 1.09% |  |  |
| #bloqueadorsolar | 31,000 | 31,300 | 0.96 | M | 0.20% |  |  |  |
| #skincancer | 30,800 | 31,100 | 0.97 | M | 0.20% |  |  |  |
| #derm | 26,900 | 27,200 | 1.11 |  | 0.18% |  |  |  |
| #tanningtips | 22,500 | 22,700 | 0.88 |  | 0.15% | 0.59% |  |  |
| #sundamage | 19,600 | 19,900 | 1.52 |  | 0.13% | 0.52% |  |  |
| #wearsunscreen | 18,100 | 18,200 | 0.55 |  | 0.12% | 0.48% |  |  |
| #selftanning | 17,300 | 17,500 | 1.15 |  | 0.11% | 0.46% |  |  |
| #tanningchallenge | 16,500 | 16,600 | 0.60 |  | 0.11% | 0.43% |  |  |
| #bestsunscreen | 15,400 | 15,700 | 1.93 |  | 0.10% | 0.41% |  |  |
| #sunscreenrecommendation | 14,200 | 14,500 | 2.09 |  | 0.09% | 0.38% |  |  |
| #cremesolaire | 14,300 | 14,400 | 0.70 | M | 0.09% | 0.38% |  |  |
| #bloqueador | n/a | 12,800 | n/a | M | 0.08% | 0.33% |  |  |
| #sunsafety | 7,083 | 7159 | 1.07 |  | 0.05% | 0.19% |  |  |
| #sunscreens | 6,133 | 6251 | 1.91 |  | 0.04% | 0.16% |  |  |
| #sunsafe | 5,156 | 5222 | 1.27 |  | 0.03% | 0.14% |  |  |
| #mineralspf | 3,284 | 3303 | 0.58 |  | 0.02% | 0.09% |  |  |
| #sunscreentest | 2,616 | 2622 | 0.23 |  | 0.02% | 0.07% |  |  |
| #sunscreenreco | 2,169 | 2201 | 1.46 |  | 0.01% | 0.06% |  |  |
| #sunlotion | 1,781 | 1806 | 1.39 |  | 0.01% | 0.05% |  |  |
| #dailyspf | 1,440 | 1460 | 1.38 |  | 0.01% | 0.04% |  |  |
| #suntanlotion | n/a | 811 | n/a |  | 0.01% | 0.02% |  |  |
|  |  |  |  |  |  |  |  |  |
|  | SUM ALL | 15,500,835 |  |  |  |  |  |  |
|  | SUM SUNSCREEN RELATED | 3,827,735 |  |  |  |  |  |  |
|  |  |  |  |  |  |  |  |  |
|  |  |  |  |  |  |  |  |  |
| Cancer-related (all <32000) |  |  |  | Trend |  |  |  |  |
| #skincancer | #melanoma |  |  | #antisunscreen | 191 |  |  |  |
| #skincancersurvivor | #carcinoma |  |  |  |  |  |  |  |
| #skincancercheck |  |  |  |  |  |  |  |  |
| #skincancerawareness |  |  |  |  |  |  |  |  |

*new hashtags that were found that did not produce high number of posts were not included in the table

**reflects decision-making of whether or not to include in the final data scrape. “Y”= yes, “M”= maybe (for consideration)
