# Supplementary material for: Sunscreen is overwhelmingly promoted on TikTok, but content with misinformation exhibits proportionally high levels of audience interaction
Source: PLOS Digit Health. 2026 Jun 18;5(6):e0001440. doi: 10.1371/journal.pdig.0001440 (PMC13278394; doi:10.1371/journal.pdig.0001440)
Supplement: S3 File — (DOCX) [file pdig.0001440.s003.docx]

**S3: Tiktok variables downloaded**

Descriptive variables:

All hashtags on tiktoks, Author ID, Author language, Author nickname , Author Signature , Author UID (numeric ID), Author unique ID, Author region, Tiktok text markup, Tiktok video ID (aweme ID), Tiktok watermark status , Tiktok share URL, Titkok title language

Tiktok Stat varibales:

number of saves/favorites (“collect count”), comments, likes, downloads, lost comments (ghost comments removed or filtered out), unsaved/unfavorite (“lose count”), plays, reposts, shares
