# Supplementary material for: Sunscreen is overwhelmingly promoted on TikTok, but content with misinformation exhibits proportionally high levels of audience interaction
Source: PLOS Digit Health. 2026 Jun 18;5(6):e0001440. doi: 10.1371/journal.pdig.0001440 (PMC13278394; doi:10.1371/journal.pdig.0001440)
Supplement: S4 File — (DOCX) [file pdig.0001440.s004.docx]

**S4: Finalized Coding Frame**

**HLI 2024**

**Coding Frame: The portrayal of sunscreen on TikTok**

**Step 1**: Read video caption before watching video (and incorporate caption into coding)

**Step 2, Inclusion**:

If the video is in English, code.

If the video is in English, and another language, and can be sufficiently interpreted, mark “MIX”, code.

If the video is not in English, mark “NE”, do not code.

**Step 3**: Code

1. Does the video’s original content contain any critiques of sunscreen (and not a specific sunscreen) related to…

1a) sunscreen preventing positive benefits from sunlight (and thus implying or explicitly that it should be avoided) (Y/N)

1b) sunscreen causing harm (either by damaging the skin or causing cancer)? (Y/N)

**(Videos that contain these messages but then are debunked by video creator do not count in this category)**

1. Does the video include images or descriptions of specific sunscreens/ a specific sunscreen (or sunscreen related product)? (Y/N)
2. Does the video contain any critiques of specific sunscreens related to health risks? (Y/N)
3. Does the video contain any critiques of specific sunscreen related to…

4a)... white casting? (Y/N)

4b)... greasy/oily feel? (Y/N)

4c)...irritating skin? (Y/N)

4d)...chemical (e.g. versus mineral)? (Y/N)

1. Does the video implicitly or explicitly state that sunscreen is valuable and should be worn? (Y/N)

5a.) Does the video debunk ideas that sunscreen should not be used or is harmful? (Y/N)

5ai) If Y in 5a, is the video a response (to comment or stitch)? (Y/N)

5b.) Does the video show the importance of solid application (e.g. a tutorial) reapplication, using everyday? (Y/N)

5c.) Does the video state or imply that sunscreen offers skin protection…

5ci) from cancer (e.g. “skin cancer,” “melanoma,” “carcinoma”)…? (Y/N)

5cii) from aging, including wrinkles (Y/N)

5ciii) from damage (general or specific rhetoric, e.g. cracking, peeling, etc.) (Y/N)

5civ) or that it helps with acne/skin beauty? (Y/N)

5cvi) Other?

1. Does the video promote a sunscreen product? (Y/N)

6a.) Does the video promote a sunscreen product by comparing or ranking sunscreens? (Y/N)

1. Does the video, including captions, include any explicit purchasing option information (e.g. links for purchasing, discount codes/special offers, etc.) ? (Y/N)
2. Does the video show alternatives/additional elements to sunscreen for sun protection (e.g. umbrellas, clothing)?
3. Is the video of a man (“M”), woman (“W”), or non-binary person (“NB”)?* [f more than one person, use central person].
4. Is the person a visible or self-described medical professional (including dermatologist)? (Y/N)

10a) Is that profession described as CAM (e.g. functional doctor, chiropractor, wellness-related, naturopath, etc.)? (Y/N)

10b) Does the video mention doctors or dermatologists? (Y/N)

1. Is the person white “W,” black “B,” Asian “A,” Middle-Eastern “ME”, or other “O”?
